# Supplementary material for: Building a better understanding of labour exploitation's impact on migrant health: An operational framework
Source: PLoS One. 2022 Aug 1;17(8):e0271890. doi: 10.1371/journal.pone.0271890 (PMC9342789; doi:10.1371/journal.pone.0271890)
Supplement: Attachment — Submitted filename: Reply to reviewers_25042022.docx [file pone.0271890.s002.docx]

25^th^ April 2022

***Response to reviewers’ comments - Manuscript Ref. No.:*** PONE-D-21-11987

Dear Editors and reviewers,

We would like to thank you very much for the very helpful peer review comments on our manuscript entitled “*Building a better understanding of labour exploitation’s impact on migrant health: An operational framework*”.

You will find our detailed replies to the reviewers’ comments and editors’ requests in Tables 1 and 2 below.

We would like to highlight that this paper describes one of the component studies of the first author’s PhD (SB) which was supervised by the co-authors, hence some of the text overlap identified by the editor. The London School of Hygiene & Tropical Medicine requires the publications of PhD thesis online, and we understand that PLoS One policies allow for submitting papers previously part of a doctoral thesis. We guarantee that this publication has not been published elsewhere.

Further, we would like to notify you that we changed the King’s College London Affiliation for the first author (SB) and replaced it by her current affiliation as ‘The University of Manchester, Humanitarian and Conflict Response Institute”. It is now modified in the manuscript.

Thank you for your interest in our manuscript.

Yours faithfully,

Dr. Sabah Boufkhed (corresponding author) and colleagues

***Table 1. Reply to reviewers’ comments***

| **Reviewers’ comments** | **Authors’ reply** |
| --- | --- |
| **Reviewer #1** | Thank you very much for your feedback and request for clarification. This has helped strengthen our manuscript. |
| Reviewer # 1 asks whether the framework applies to international labor migrants only - non inclusive of internal migrant workers in exploitative conditions. Is this correct? | Thank you for your feedback. The framework was indeed designed for international labour migrants as a first step. Further research would be needed to assess whether the framework could be used in internal migration context.  We clarified this in the core text after the definition we use for ‘migrant workers’, as follows  “This paper will focus on international migrant workers.” (line 56) |
| Further, does the proposed framework of Labour exploitation consider the phases of migrant cycle? The model needs to better consider/integrate/enshrine exploitation not just at the point of destination but also at point of origin prior to departure. Exploitation occurs at country of origin across migration (mis)information, recruitment phases, mandatory health examinations as pre-requisite for labour migration, etc. The exploitation also occurs post-arrival phases for some migrant workers and manifests in forms such as non provision of social welfare support, health and legal protections upon return. | Thank you for your comment. The framework has indeed considered the phases of migrant cycle, and we have now clarified this in the discussion as follows:  “The current research was designed within a pragmatic epistemology that aimed to facilitate the understanding of labour exploitation in the labour market of the ‘destination’ or ‘host’ country, with the objective to measure and identify labour exploitation where the international migrants work. We acknowledge that international migrant workers’ exploitation may occur differently at different stages of the migration cycle [92], and our framework may not account for situation in the pre- or post-destination migration phases.” (lines 433-38) |
| As authors indicate non-random sampling method used may limit the generalizability of the findings as participating experts may have similar political leanings towards the topic and were mainly from high-income countries - with half of the sample being from the UK. Regions such as the Gulf Cooperation Council (GCC) which is one of the main destination regions globally for international migrant workers, and where proportion of migrant to local workers is amongst the highest in the world. Unclear as to the representation from this region. | Thank you for this comment.  During the identification of experts, we mapped out experts from and of every region of the world. Unfortunately, we did not have responses from all regions as mentioned in the discussion.  While we were not able to recruit experts from every region of the world, we did however recruit experts with broad and international expertise on labour exploitation. We believe that the statements generated would be widely applicable. The framework’s novelty is that it offers a tool to capture labour exploitation at the destination country in a holistic and multidimensional way, with items connected to structural aspects of exploitation (e.g. the social and legal dimension) and the aspects connected to management of workers (e.g. exposure to violence).  For example, the Kafala system that is still implemented in several countries in GCC would result in situations captured in several dimensions of labour exploitation described in the framework, such as:  - the lack of Social and Legal Protection (e.g. “s/he can be dismissed at will”; s/he does not benefit from social protection benefits; “s/he is treated worse than the legally acceptable minimum in the country where s/he works”)  - Health and Safety issues (e.g. “s/he works in unsafe conditions”)  - Issues related to Finance and Migration (e.g. “his/her working permit is linked to the current employer”; s/he is lied to about his/her rights as a migrant in the country where s/he is working; s/he is paid below the wage of national workers for the same job)  - Issues related to Shelter and Personal security (e.g. s/he is threatened with deportation”; “s/he is obliged to work under cruel or inhumane conditions”)  The framework’s novelty is that it offers a tool to capture labour exploitation at the destination country in a holistic and multidimensional way, with items connected to structural aspects of exploitation (e.g. the social and legal dimension) and the aspects connected to management of workers (e.g. exposure to violence). The framework provides a much-needed basis for future research that could test whether it is applicable to GCC countries.  We added the following specification in the discussion:  “While we identified experts from various regions, we were not able to recruit experts from every region of the world. We did however recruit experts with a broad and international expertise on labour exploitation. We believe that the statements generated would be widely applicable. The framework’s novelty is that it offers a tool to capture labour exploitation at the destination country in a holistic and multidimensional way, with items connected to structural aspects of exploitation (e.g. the social and legal dimension) and the aspects connected to management of workers (e.g. exposure to violence).” (lines 451-57) |
| **Reviewer #2** |  |
| The second reviewer raises the following (minor) issues. Please address these and revise your manuscript accordingly: | Thank you very much for the positive feedback and supportive comments. |
| Lines 218-220 state that a second concept map was produced by migrant workers. It was not clear to me if this was included in the results or if it will be analyzed/published separately, but I would like to see more discussion of this in the paper and would be interested to know how it complemented/differed from the other concept map. | Thank you very much for highlighting this point. We indeed performed a Concept Mapping (GCM) with migrant workers. The GCM method was adapted for the population that is considered vulnerable and hard-to-reach, and was conducted in person. Given the word limitations and the difference in method, we could not include it as part of one paper. It will constitute a stand-alone paper that would bring the voices of migrant workers. In order to avoid confusion for the reader, we adapted the lines mentioned by reviewer #2 as follows:  “Migrant workers are also considered experts on the topic [74], and we have undertaken further research with international migrants working in low skilled jobs in London to assess their conceptualisation [19].” (lines 209-11) |
| The framework proposed by Cwikel (line 170) needs a reference | Thank you very much for pointing this out.  We have now amended the references. |
| Line 230: there is a possible grammatical error, as the line does not make sense to reviewer #2. | Thank you very much for pointing this out, there are indeed few words missing. We amended as follows:  “It is expected that the statements generated for the sorting-rating exercise are varied enough to cover all aspects of the concept to be mapped***,*** and it is common ***to*** include ***new participants*** who did not participate in the first phase (3,4).” (lines 221-22) |

***Table 2. Reply to editor’s requirements for publication***

| **Editor’s requirements** | **Authors’ reply** |
| --- | --- |
| 1. Please ensure that your manuscript meets PLOS ONE's style requirements, including those for file naming. The PLOS ONE style templates can be found at  <https://journals.plos.org/plosone/s/file?id=wjVg/PLOSOne_formatting_sample_main_body.pdf> and  <https://journals.plos.org/plosone/s/file?id=ba62/PLOSOne_formatting_sample_title_authors_affiliations.pdf> | Thank you, we updated the title page and the in-text citation to correspond to the criteria. |
| 2. In your Data Availability statement, you have not specified where the minimal data set underlying the results described in your manuscript can be found. PLOS defines a study's minimal data set as the underlying data used to reach the conclusions drawn in the manuscript and any additional data required to replicate the reported study findings in their entirety. All PLOS journals require that the minimal data set be made fully available. For more information about our data policy, please see <http://journals.plos.org/plosone/s/data-availability>  Upon re-submitting your revised manuscript, please upload your study’s minimal underlying data set as either Supporting Information files or to a stable, public repository and include the relevant URLs, DOIs, or accession numbers within your revised cover letter. For a list of acceptable repositories, please see http://journals.plos.org/plosone/s/data-availability#loc-recommended-repositories. Any potentially identifying patient information must be fully anonymized.  Important: If there are ethical or legal restrictions to sharing your data publicly, please explain these restrictions in detail. Please see our guidelines for more information on what we consider unacceptable restrictions to publicly sharing data: http://journals.plos.org/plosone/s/data-availability#loc-unacceptable-data-access-restrictions. Note that it is not acceptable for the authors to be the sole named individuals responsible for ensuring data access. | The dataset used is now provided as a Supporting Information file 1 that we uploaded (matrix for the multivariate analysis).  We also added the following specification at the end of the manuscript as recommended in the PLoS One guidelines:  “Supporting information  **S1 File. Dataset for the multivariate and cluster analyses.** Legend: The numbers in columns and rows correspond to the number of statements’ ID numbers as identified in Table 2 and mapped on Fig 2.” |
| 3. We note that you have stated that you will provide repository information for your data at acceptance. Should your manuscript be accepted for publication, we will hold it until you provide the relevant accession numbers or DOIs necessary to access your data. If you wish to make changes to your Data Availability statement, please describe these changes in your cover letter and we will update your Data Availability statement to reflect the information you provide. | Please see reply above |
| 4. Your abstract cannot contain citations. Please only include citations in the body text of the manuscript, and ensure that they remain in ascending numerical order on first mention. | N/A |
| 5. Please include your full ethics statement in the ‘Methods’ section of your manuscript file. In your statement, please include the full name of the IRB or ethics committee who approved or waived your study, as well as whether or not you obtained informed written or verbal consent. If consent was waived for your study, please include this information in your statement as well. | The ethical statement was added in the Method section: lines 225-28 |
| 6. Thank you for submitting the above manuscript to PLOS ONE. During our internal evaluation of the manuscript, we found significant text overlap between your submission and the following previously published works, some of which you are an author.  - <https://researchonline.lshtm.ac.uk/id/eprint/4656024/1/2019_PHP_PhD_Boufkhed_S-Copy.pdf>  We would like to make you aware that copying extracts from previous publications, especially outside the methods section, word-for-word is unacceptable. In addition, the reproduction of text from published reports has implications for the copyright that may apply to the publications.  Please revise the manuscript to rephrase the duplicated text, cite your sources, and provide details as to how the current manuscript advances on previous work. Please note that further consideration is dependent on the submission of a manuscript that addresses these concerns about the overlap in text with published work.  We will carefully review your manuscript upon resubmission, so please ensure that your revision is thorough. Please pay special attention to the sections of the results that have overlap. | We would like to emphasise that this paper describes one of the component studies of the first author’s PhD thesis (<https://researchonline.lshtm.ac.uk/id/eprint/4656024/1/2019_PHP_PhD_Boufkhed_S-Copy.pdf>) which was supervised by the co-authors. The London School of Hygiene & Tropical Medicine requires the publications of PhD thesis online under the ‘Creative Commons: Attribution-Noncommercial-No Derivative Works 3.0 licence’. We have run the manuscript through Turnitin software and the vast majority of text overlap is with the thesis. We understand that PlosOne policies allow for submitting papers that were previously part of a doctoral thesis and guarantee that this publication has not been published elsewhere.  We have rephrased where possible, however it is unavoidable that significant pieces would remain similar.  The majority of the similarity text comes from the theory that was developed in the thesis (the two schools of thoughts name and description; the discussion on terminology used for labour exploitation), name of reports or special issues meaningful for the argumentation. and direct quotes from the findings (e.g. many statements generated as part of the concept mapping exercise) which cannot be rephrased without changing the meaning of the work.  Most of the remaining overlap text is mostly short phrases (e.g. statements of facts, sentence openers). To deal with this we have edited those sections which can be edited to use forms of word which differ from those in the thesis.  *In the introduction:*  The vast majority of the overlap text is the description of a new categorisation of the two schools of thoughts on labour exploitation that was never discussed in the literature before. It may not be meaningfully rephrased without changing the meaning and description.  *In the methods:*  We have also now more explicitly acknowledged and cited the PhD thesis in the methods:  “The methodology is detailed in full in the doctoral thesis from which this research is from [19].” (line 187-88)  Please note that we also mentioned the thesis in the Introduction:   - “This study **formed part of the first author's doctoral thesis**, which [19] aimed to develop an operational conceptual framework of labour exploitation focusing on migrant workers in manual low-skilled jobs, building on a continuum approach.” (lines 68-70) - “The larger research project **(SB’s doctoral thesis [19])** within which this study is nested addresses the first phase:” (lines 164-65)   *In the Findings:*  The overlap text corresponds to the description of the population (e.g. percentage of academic and non-academic professionals) and the actual findings (e.g. names of the dimensions, subdimensions, items, tables’ content describing the framework).  *In the discussion:*  Most of the overlap is from extract of items described in the findings and results table, and from short statements such as opener and stating facts that are referenced. |
